# Parents' perception of their children's neurodevelopment during the COVID-19 pandemic and associated factors

**DOI:** 10.1016/j.jped.2025.06.002

**Published:** 2025-07-16

**Authors:** Sabrina Ribeiro Ibiapina, Taís Michele Werle, Milena Monticelli Giongo, Magda Lahorgue Nunes

**Affiliations:** a Pontifícia Universidade Católica do Rio Grande do Sul (PUCRS), Programa de Pós-Graduação em Pediatria e Saúde Infantil, Porto Alegre, RS, Brazil; b Pontifícia Universidade Católica do Rio Grande do Sul (PUCRS), Escola de Medicina, Programa de Bolsa de Pesquisa de Júnior de Graduação, Porto Alegre, RS, Brazil; c Pontifícia Universidade Católica do Rio Grande do Sul (PUCRS), Escola de Medicina, Porto Alegre, RS, Brazil; d Pontifícia Universidade Católica do Rio Grande do Sul (PUCRS), Instituto do Cérebro, Porto Alegre, RS, Brazil

**Keywords:** COVID-19 pandemic, Children, Neurodevelopmental disorders, Parents, Perception

## Abstract

**Objective:**

To verify parents’/caregivers’ perceptions of changes in their children’s development during the COVID-19 pandemic and factors associated with such perceptions.

**Methods:**

Cross-sectional study using an online survey made available to parents/caregivers of children between 0-7 years old, from September 2021 to March 2023 in two Brazilian states. Respondents answered questions about their perceptions regarding their children's neurodevelopment, and worsening of neurodevelopment during the COVID-19 pandemic. Validated questionnaires such as M-CHAT, “Swanson, Nolan and Pellham,” and “Strengths and Difficulties “were applied according to age. Comparisons were made using the chi-square test or Fisher's exact test, and Poisson regression was used in the univariate analysis and in the multivariate analysis.

**Results:**

Data from 589 children were obtained, 49.7% aged 0-3 years and 50.3% 4-7 years. Of the 0-3 age group, 50 (17.1%) were perceived as having abnormal neurodevelopment, and 79 (27.0%) as having worsened neurodevelopment during the pandemic. Of the 4-7 year group, 76 (25.7%) were perceived as having abnormal neurodevelopment, and 104 (35.1%) as having worsened their neurodevelopment. Significant risk factors associated with the perception of abnormal neurodevelopment were maternal schooling, the child's sex and age; for the perception of worsening neurodevelopment were the child's sex and age, low socioeconomic status, degree of social isolation, and death in the family due to COVID-19.

**Conclusion:**

Data from the present study showed that parents/ caregivers' perception of normal neurodevelopment was significantly higher than their recognition of abnormalities. In addition, a significant percentage perceived a worsening during the COVID-19 pandemic.

## Introduction

The COVID-19 pandemic, which occurred between 2020 and 2021, has affected the pediatric population in many ways. The impact began with the interruption and limitation of social interaction, including the closure of schools, leading to a change in routine with intense home confinement, culminating in various behavioral, psychological and neurodevelopmental repercussions.^1^, ^2^, ^3^, ^4^

Several studies were published during and after the pandemic, showing an increase in sleep problems in children and adolescents, an impact on relationships due to the deprivation of social interaction with peers, as well as limited opportunities for physical and creative activities. In addition, they report behavioral changes and mood swings in children, especially those diagnosed with ASD (autism spectrum disorder).^5^, ^6^, ^7^

Mental health in the pediatric population has become more visible as a result of the changes identified. Studies suggest an increase in neurobehavioral disorders, such as associations with agitation, restlessness, and difficulty with attention/concentration, emotional problems, aggressive behavior, and an impact on social skills. Another aspect that has been widely observed and referenced is the increase in the time children are exposed to and use electronic devices, as well as sleep problems during home confinement.^3^^,^^4^^,^^8^, ^9^, ^10^

Due to the findings previously described, the aim of this study was to verify parents’/caregivers’ perceptions of changes in their children’s development during the COVID-19 pandemic and factors associated with such perceptions. A secondary aim was to compare among states eventual differences in this perception.

The authors hypothesized that differences between perception of worsening and perception of abnormality in the neurodevelopment of children during the COVID-19 pandemic might occur.

## Methods

### Type of study and data collection

This is a cross-sectional observational study with a target population of children aged 0-7 years, with the participants/respondents of the research being the parents/caregivers of this target population. The research was conducted in the city of Porto Alegre, located in the state of Rio Grande do Sul (RS), in southern Brazil, and in the city of Fortaleza, located in the state of Ceará (CE), in northeastern Brazil. These states were chosen for convenience and because both had adopted preventive and restrictive measures as a contingency plan to deal with the pandemic, which were widely followed by the population.^11^^,^^12^

The data were collected using an online questionnaire made available by the digital data collection platform (online survey software) Qualtrics® (www.qualtrics.com). The online format was chosen in order to collect data simultaneously in the two different cities. This study was approved by the Research Ethics Committee of the Pontifical Catholic University of Rio Grande do Sul (PUCRS) and is registered on the Brazil Platform under the number 47217921.2.0000.5336.

Respondents were invited to participate by advertising the survey on the digital social media of the Pontifical Catholic University of Rio Grande do Sul and the Brain Institute of Rio Grande do Sul, radio spots and local/state television, as well as posters and folders with QR codes and links to the survey displayed in schools and pediatric clinics in the city of Porto Alegre. An active search for respondents was also carried out, with the questionnaire being applied in person in pediatric care settings linked to the PUCRS School of Medicine (Basic Health Units and the Hospital São Lucas PUCRS Clinical Center). In Fortaleza, dissemination also took place via social media, with the access link being sent out, as well as posters and folders with QR codes displayed in public and private pediatric clinics and institutions. The active search was conducted in basic health units, where electronic devices were made available on site directly by the researchers themselves. The questionnaire was open for responses between September 2021 and March 2023, with no request for respondents to access the survey again.

### Protocols and scales

Consent was obtained by means of a form (Informed Consent Form - ICF) applied at the beginning of the online questionnaire, after a brief descriptive explanation of the functioning and purpose of this study. Respondents could only proceed with the survey, accessing the questions in the questionnaire, after agreeing to take part in the study and agreeing to the online ICF by clicking on "I agree to take part in this study". If they did not agree to take part, they were directed to the thank-you page.

After accepting and agreeing, the respondent was initially directed to collect sociodemographic data, followed by questions related to the history and course of pregnancy, prenatal care, childbirth, the neonatal period, and the child's health. In the sequence come questions related to neurodevelopment, according to the age group. First, the respondents state their perception regarding their children’s neurodevelopment compared to children of the same age group, and also whether they have perceived a worsening in their children’s neurodevelopment during the COVID-19 pandemic. The variables related to the assessment of neurodevelopment were based on the Denver Scale II, which was adapted into the form of a questionnaire, and on the Child Development Form of the Early Childhood for Healthy Adults program (PIPAS).^13^, ^14^, ^15^, ^16^

The interviewees were then directed to specific questionnaires on behavioral and neurodevelopmental disorders, all previously validated for Brazilian Portuguese. Given that early signs of autism spectrum disorder (ASD) can be identified in young children, the “Modified Checklist for Autism in Toddlers” (M-CHAT) scale was used, which is recommended for children between 18 and 24 months of age. According to the total score of the M-CHAT, children were classified as low risk for ASD (0-2 points); moderate risk (3-6 points), and high risk (7-23 points) for ASD.^17^, ^18^, ^19^, ^20^, ^21^

The MTA-SNAP IV scale (shortened version of the Swanson, Nolan and Pellham Questionnaire) was used to assess the symptoms of attention deficit hyperactivity disorder (ADHD) in children aged 4 and older, the recommended age for such screening. It consists of 26 items divided into three subscales: inattention, hyperactivity/impulsivity and oppositional/challenging behavior. The score for each subscale was added together to generate a raw score according to the following classifications: clinically non-significant symptoms (< 13 points); mild symptoms (13-17 points); moderate symptoms (18-22 points); severe symptoms (23-27 points).^22^, ^23^, ^24^

To assess and screen for mental health problems in children aged 4 and over, the authors used the "Strengths and Difficulties Questionnaire" (SDQ) - parent version. This version consists of 25 items distributed in 5 subscales (emotional symptoms, conduct problems, hyperactivity, relationship problems with peers, and pro-social behavior). The SDQ was classified according to the total difficulty score as normal (0-13 points), borderline (14-16 points), and abnormal (17-40 points).^25^^,^^26^

The last battery of questions referred to the situation of restrictions related to the COVID-19 pandemic in the respondent's city, and also how the family behaved during the pandemic period, as well as addressing the use of electronic devices and the time the child was exposed to screens through two specific questions. At the end of the questionnaire, an automatic message was generated confirming that the questionnaire had been completed and thanking them for taking part in the survey, along with an information table on warning signs of neurodevelopmental problems. For data analysis, the authors only included data from respondents who completed the questionnaire completely and to the end.

### Sample calculation and statistical analysis

A total sample size of 550 children was calculated to test whether there is a difference between the percentages of perception of worsening and perception of abnormality in the neurodevelopment of children during the COVID-19 pandemic between the groups, generated from the classifications of various characteristics under study (including income, maternal education, maternal age, sex of the child, among others). The calculation considered groups of similar size, power of 80%, significance level of 5%, baseline percentage of perception of worsening or abnormality of 22% and prevalence ratio of at least 1.5 as the minimum relevant effect.

The values observed for the two outcomes considered (perception of worsening and perception of abnormality in neurodevelopment) were described by count and percentages between the groups formed by the classifying variables, including their distribution among the states in the sample. All initial comparisons were carried out using the Chi-square test or Fisher's exact test, when necessary.

Using Poisson regression, the prevalence ratio of perception of worsening or abnormality in neurodevelopment, its 95% confidence interval, and the p-value were obtained for each classifying variable. All the variables that showed p < 0.20 in the univariate analysis were included in a multivariable Poisson model to adjust the effects, obtaining the same statistics as above.

The respondents' perceptions of worsening and abnormality in neurodevelopment were compared with the reference values of the selected screening scales, M-CHAT, SDQ and SNAP. This resulted in sensitivity rates and false positive rates. Sensitivity rates indicated the extent to which respondents reported perceiving worsening and/or abnormality in the neurodevelopment of children classified as moderate/high risk (or similar denomination) by the selected scale. False positive rates indicate the extent to which respondents reported perceiving worsening and/or abnormality in the neurodevelopment of children classified as low risk (or similar denomination) by the selected scale.

All statistical procedures were carried out using the IBM-SPSS version 25.0 program. Findings with a value of P<0.05 were considered statistically significant.

## Results

In total, the survey had 496 respondents, 220 from RS and 276 from CE. The survey thus resulted in 589 children, 271 from RS and 318 from CE, as each respondent could include more than one child during the course of answering the questionnaire.

Table 1 shows the sociodemographic data of both the respondents and the children. In both states, the survey respondent was predominantly the child's mother and there was a significant difference between the states (p < 0.001), showing that mothers in RS were younger, and their schooling was lower (p < 0.001). With regard to self-reported monthly income, in CE there was a predominance of higher incomes among the respondents (p < 0.001), and in RS there was a predominance of Social Program participants (p = 0.012). Emphasizing the pandemic context, in the CE, there was greater compliance with local guidelines for social isolation, with a significant difference between the groups (p = 0.001). The reported cases of COVID-19 diagnoses in the family were higher in CE than in RS, (p = 0.001), as were the cases of COVID-19 deaths in family members (p = 0.019). With regard to children, there were statistically significant differences in the following variables: gender (p = 0.001), with a predominance of males in CE (61.0%) and females in RS (52.8%); age group (p = 0.005), with older children predominating in CE; and schooling (p < 0.001), with a higher number of children who were not attending school in RS.

**Table 1:** Demographic data of the respondents and the children under study.

Variable	CE	RS	P
**Respondents**	**(N = 276)**	**(N = 220)**	
Who answered the questionnaire, no. (%)			0.694
Mother	253 (91.7)	200 (90.9)	
Dad	12 (4.3)	8 (3.6)	
Other	11 (4.0)	12 (5.5)	
Maternal age in years, no. (%)			0.173
< 18	5 (1.8)	9 (4.1)	
≥ 18	271 (98.2)	211 (95.9)	
Median (AIQ)	37 (32-40)	33 (25-38)	**<0.001**
Mother's schooling, number/total number (%)			**<0.001**
Elementary School	7/276 (2.5)	34/202 (16.8)	
MS up to incomplete degree	52/276 (18.8)	82/202 (40.6)	
Full degree and PG	217/276 (78.6)	86/202 (42.6)	
Who the child lives with, no. (%)			**<0.001**
Responsible 1 and 2	214 (77.5)	152 (69.1)	
Responsible 1 (father or mother)	31 (11.2)	11 (5.0)	
Responsible 2 (father or mother)	25 (9.1)	46 (20.9)	
Another family member	6 (2.2)	11 (5.0)	
Family income in minimum wages, no. (%)			**<0.001**
Up to 1	30 (10.9)	68 (30.9)	
Between 1 and 2	29 (10.5)	37 (16.8)	
From 2 to 4	34 (12.3)	36 (16.4)	
From 4 to 10	74 (26.8)	36 (16.4)	
From 10 to 20	71 (25.7)	22 (10.0)	
Above 20	38 (13.8)	21 (9.5)	
Participates in Social Program, no. (%)	39 (14.1)	51 (23.3)	**0.013**
Did social isolation (*), number/total number (%)	255/275 (92.4)	186/218 (85.3)	**0.011**
COVID in the family, number/total number (%)	204/269 (73.9)	136/217 (62.7)	**0.002**
Death due to COVID in the family, no. (%)	31 (11.2)	13 (5.9)	**0.040**
**Children**	**(N=318)**	**(N=271)**	
Sex, number (%)			**0.001**
Female	124 (39.0)	143 (52.8)	
Male	194 (61.0)	128 (47.2)	
Age group, number (%)			**0.005**
0-3 years	142 (44.7)	151 (55.7)	
4-7 years	176 (55.3)	120 (44.3)	
Schooling, number/total number (%)			**<0.001**
They don't study	60/316 (18.9)	110/271 (40.6)	
Nursery	74/316 (23.3)	78/271 (28.8)	
Pre-school	113/316 (35.5)	47/271 (17.3)	
1st grade Elementary School	36/316 (11.3)	20/271 (7.4)	
2nd grade Elementary School	26/316 (8.2)	15/271 (5.5)	
3rd grade Elementary School	7/316 (2.2)	1/271 (0.4)	

CE, State of Ceará; RS, State of Rio Grande do Sul; P, statistical significance; AIQ, interquartile range. (*) According to the regulations in force in the region. COVID, disease caused by the coronavirus.

Of the 589 children, 293 (49.7%) were in the 0-3 years age group and 296 (50.3%) were in the 4-7 years age group. Among the children aged 0 to 3, respondents perceived 50 (17.1%), as having abnormal neurodevelopment, and 79 (27.0%) worsened their neurodevelopment during the pandemic. Among children aged 4 – 7, 76 (25.7%) were initially perceived as having abnormal neurodevelopment, and in 104 (35.1%) responders perceived a worsening on neurodevelopment worsened during the COVID-19 pandemic.

Comparing both states (RS and CE) and both age groups, there was an increased perception of abnormal neurodevelopment and worsening neurodevelopment during the pandemic in the age group 4-7. Regarding states, this perception of CE was significantly higher than in RS (p < 0.001).

Table 2 shows the univariate and multivariable regression analyses regarding the perception of worsening in children's neurodevelopment during the COVID-19 pandemic. In the univariate analysis, significance was observed in relation to the variables family income, maternal schooling, sex of the child, age group of the child, having been in social isolation, and cases of death from COVID-19 in the family. After adjustment, the variables maternal schooling, sex of the child and age group of the child remained significant, proving to be risk factors associated with the outcome of perception of worsening neurodevelopment during the COVID-19 pandemic.

**Table 2:** Respondents' perception of worsening neurodevelopment during the COVID-19 pandemic.

				Univariate analysis			Multivariable analysis*		
Features	N	no.	%	PR	95% CI	P	PR	95% CI	P
Family income									
up to 2 MW	202	54	26.7	Ref.	–	–	Ref.	–	–
2 to 10 MW	207	64	30.9	1.14	0.83 – 1.56	0.412	0.76	0.50 – 1.15	0.198
> 10 MW	180	65	36.1	1.31	0.96 – 1.79	0.086	0.76	0.48 – 1.21	0.248
Education									
Fundamental	50	9	18.0	Ref.	–	–	Ref.	–	–
Up to incomplete degree	172	48	27.9	1.51	0.76 – 2.99	0.236	1.51	0.78 – 2.93	0.224
Undergraduate and PG	344	124	36.0	1.92	1.00 – 3.68	0.050	2.22	1.05 – 4.69	**0.036**
Maternal age									
up to 18 years old	16	4	25.0	0.84	0.36 – 2.00	0.701			
> 18 years	573	179	31.2	Ref.	–	–			
Sex of the child									
Female	267	69	25.8	0.75	0.59 – 0.96	0.022	0.74	0.58 – 0.94	**0.013**
Male	322	114	35.4	Ref.	–	–	Ref.	–	–
Child's age group									
0 to 3 years	293	79	27.0	Ref.	–	–	Ref.	–	–
4 to 7 years	296	104	35.1	1.36	1.07 – 1.74	0.012	1.34	1.05 – 1.72	**0.020**
Participation Social program									
Yes	113	36	31.9	1.04	0.76 – 1.43	0.800			
No	476	147	30.9	Ref.	–	–			
Social isolation									
Yes	522	169	32.4	1.59	0.94 – 2.70	0.085	1.36	0.81 – 2.28	0.241
No	67	14	20.9	Ref.	–	–	Ref.	–	–
COVID-19 in the family									
Yes	397	127	32.0	1.08	0.82 – 1.41	0.596			
No	192	56	29.2	Ref.	–	–			
Death from COVID-19 in the family									
Yes	49	20	40.8	1.29	0.88 – 1.89	0.185	1.35	0.92 – 1.99	0.121
No	540	163	30.2	Ref.	–	–	Ref.	–	–

COVID- 19, disease caused by the coronavirus; N, total; no., perception of worsening; PR, prevalence ratio; CI, confidence interval; P, statistical significance; Ref, reference group; MW, minimum wage; HS, high school; PG, postgraduate; (*) all variables with a p-value < 0.20 in any of their categories were included in the multivariable model.

Table 3 shows the univariate and multivariable regression analyses regarding the perception of abnormal neurodevelopment compared. In the univariate analysis, the variables family income, gender of the child, age group of the child, participation in a Social Program, having been in social isolation and cases of death from COVID-19 in the family reached a statistical significance. In the multivariable analysis, after adjustment, the variables sex of the child, age group of the child, participation in a Social Program, having been socially isolated and cases of death by COVID-19 in the family remained significant.

**Table 3:** Perception of neurodevelopmental abnormality by respondents during the COVID-19 pandemic.

				Univariate analysis			Multivariable analysis*		
Features	N	no.	(%)	PR	95% CI	P	PR	95% CI	P
Family income									
up to 2 MW	202	52	25.7	Ref.	–	–	Ref.	–	–
2 to 10 MW	207	32	15.5	0.60	0.41 – 0.89	0.011	0.86	0.56 – 1.34	0.514
> 10 MW	180	42	23.3	0.91	0.63 – 1.30	0.589	1.32	0.83 – 2.09	0.247
Education									
Fundamental	50	9	18.0	Ref.	–	–			
Up to incomplete degree	172	40	23.3	1.27	0.63 – 2.59	0.507			
Undergraduate and PG	344	74	21.5	1.19	0.60 – 2.36	0.626			
Maternal age									
up to 18 years old	16	4	25.0	1.18	0.48 – 2.89	0.714			
> 18 years	573	122	21.3	Ref.	–	–			
Sex of the child									
Female	267	37	13.9	0.50	0.35 – 0.71	<0.001	0.51	0.36 – 0.71	**<0.001**
Male	322	89	27.6	Ref.	–	–	Ref.	–	–
Child's age group									
0 to 3 years	293	50	17.1	Ref.	–	–	Ref.	–	–
4 to 7 years	296	76	25.7	1.51	1.09 – 2.08	0.013	1.50	1.10 – 2.05	**0.012**
Participation Social program									
Yes	113	40	35.4	1.96	1.43 – 2.69	<0.001	2.06	1.35 – 3.13	**0.001**
No	476	86	18.1	Ref.	–	–	Ref.	–	–
Social isolation									
Yes	67	8	11.9	1.89	0.98 – 3.64	0.056	1.93	1.04 – 3.60	**0.039**
No	522	118	22.6	Ref.	–	–	Ref.	–	–
COVID-19 in the family									
Yes	397	79	19.9	0.81	0.59 – 1.12	0.200			
No	192	47	24.5	Ref.	–	–			
Death from COVID-19 in the family									
Yes	49	17	34.7	1.72	1.14 – 2.60	0.009	1.89	1.28 – 2.79	**0.001**
No	540	109	20.2	Ref.	–	–	Ref.	–	–

COVID-19, disease caused by the coronavirus; N, total; no., perception of neurodevelopmental abnormality; PR, prevalence ratio; CI, confidence interval; P, statistical significance; Ref, reference group; MW, minimum wage; HS, high school; PG, postgraduate; (*) all variables with a p-value < 0.20 in any of their categories were included in the multivariable model.

According to Table 4, the respondents generally agreed more with the normal/low risk classifications of the scales used. The greatest predominance of agreement between perception of abnormality and risk classification was observed in the Inattention subscale of the SNAP scale, moderate/severe signs (68.6%). As for the respondents' perception of worsening in children's neurodevelopment during the pandemic, agreement was higher with the risk classification, also of the Inattention subscale of the SNAP scale, moderate/severe signs (77.1%).

**Table 4:** Comparison between respondents’ perceptions and ratings by the selected scales.

Perceptions – Scales	Total
Perceptions – M-CHAT, no./total no. (%)	(N = 79)
Abnormality – Moderate, hight risk	8/31 (25.8)
Normality – Low risk	44/48 (91.7)
Worsening in the pandemic – Moderate, hight risk	10/31 (32.3)
Perceptions – SDQ, no./total no. (%)	(N=296)
Abnormality – Abnormal	52/153 (34.0)
Normality – Normal, boderline	119/143 (83.2)
Worsening in the pandemic – Abnormal	64/153 (41.8)
Perceptions – SNAP (Inattention), no./total no. (%)	(N=296)
Abnormality – Moderate, severe	24/35 (68.6)
Normality – No signs, mild signs	209/261 (80.1)
Worsening in the pandemic – Moderate, severe	27/35 (77.1)
Perceptions – SNAP (Hyperactivity, impulsivity), no./total no. (%)	(N=296)
Abnormality – Moderate, severe	14/39 (35.9)
Normality – No signs, mild signs	195/257 (75.9)
Worsening in the pandemic – Moderate, severe	19/39 (48.7)
Perceptions – SNAP (Oppositional, challenging behavior), no./total no. (%)	(N=296)
Abnormality – Moderate, severe	5/23 (21.7)
Normality – No signs, mild signs	202/273 (74.0)
Worsening in the pandemic – Moderate, severe	6/23 (26.1)

M-CHAT, Modified Checklist for Autism in Toddlers scale; SDQ, Multimodal Treatment Study of Children with Attention-Deficit Hyperactivity Disorder scale; SNAP, Strenghs and Difficulties Questionnaire scale; no., respondents’ perceptions; total no., classification by the scale; N, total number of children covered by the scale.

In addition, the sensitivity rate and the false positive rate of the comparisons between the respondents' perceptions and the scale ratings were calculate the highest sensitivity rate was obtained between the SNAP (Inattention) scale and respondents' perceptions of abnormal neurodevelopment (68.6%) and worsening neurodevelopment during the pandemic (77.1%). In other words, the respondents showed a perception of abnormality and worsening in development in children who were classified as at risk by this scale. The lowest false positive rate was obtained between the M-CHAT scale and respondents' perceptions of abnormal neurodevelopment (8.3%) and worsening neurodevelopment during the pandemic (16.7%). This means that respondents reported perceiving abnormal neurodevelopment and worsening neurodevelopment in children who were classified by the scale as low risk.

## Discussion

This study provides information about the perceptions of parents and/or caregivers regarding the neurodevelopment of their children during the COVID-19 pandemic and the risk factors associated with such perceptions. It also presents the sensitivity rates and false positive rates of these perceptions in relation to the results of screening scales for neurodevelopmental disorders.

In general, the respondents' perception of normal neurodevelopment is higher than their recognition of abnormal neurodevelopment. Regarding the perception of worsening neurodevelopment during the COVID-19 pandemic, this was reported in both states, although there was a greater predominance of this perception in the 4-7 years age group. Due to the impact of the COVID pandemic in many aspects of life, this topic has also been investigated in other countries, with some studies applying similar methodology.

A cross-sectional study carried out in Turkey assessed psychological and behavioral changes in children at different ages (early childhood, n = 249; second childhood, n = 193; and adolescence, n = 399) before and during the COVID-19 pandemic, from the perspective of their parents' perceptions, using an online questionnaire. The study found that parents predominantly perceived sleep-related problems in early childhood, behavioral changes and psychological symptoms in later childhood, and behavioral and psycho-emotional changes, as well as cognitive changes in adolescents.^27^

In a cohort study carried out in Japan, with a sample of 887 children, 447 in the 1-3 years group and 440 in the 3-5 years group, the perception of negative impact varied. In the 1-3 age group, the exposed cohort did not show a negative association between the pandemic and general development. In contrast, in the 3-5 age group, children were 4.39 months behind in overall development at age 5 compared to controls, in all subdomains (broad motor, fine motor, receptive language, expressive language, language concepts, social relationships with children, social relationships with adults, and discipline).^28^ Similarly, in the present study, the greatest impacts were reported in older children, aged 4-7.

A Brazilian cross-sectional study analyzed, through the perception of family members who answered an online questionnaire, the effects of a lack of school environment during the pandemic on the development of their children aged between 1-5 years. Results showed that the socio-emotional aspect was the most negatively affected (this perception was more intense in the group of children over three years old), followed by the cognitive-linguistic aspect. Some aspects were mentioned as having had a positive impact, with the cognitive-linguistic aspect being perceived as more positive than the motor and socio-emotional aspects; however, it should be noted that the number of respondents who did not identify positive effects was higher than those who did not perceive negative effects. However, in the perception of the respondents, with regard to child development, the negative impacts of the pandemic were greater than the positive ones, regardless of the age group assessed.^29^

A cross-sectional study carried out in the United States involving 37 subjects (health professionals, childcare providers and parents), showed their concern about the limitations of the pandemic impacting social interactions, the lack of access to peer models, and fewer opportunities for physical exploration and creative activities to promote child development.^7^

In order to demonstrate these impacts on development and with an emphasis on the first year of life, a cohort study carried out in the United States examined the neurodevelopment of babies born during the pandemic, at birth, and at 6 months of age, children of mothers infected with and mothers not infected with SARS-CoV-2. Using the Ages & Stages Questionnaire 3rd Edition (ASQ-3) protocol, it was found that in utero exposure to maternal SARS-CoV-2 infection was not associated with significant differences in any ASQ-3 subdomain. However, compared to the historical cohort, infants born during the pandemic had significantly lower scores in the domains of broad and fine motor skills and personal-social skills at 06 months of age.^30^

Further, a systematic review and meta-analysis evaluated the association of birth and development during the COVID-19 pandemic found an increased risk of impaired neurodevelopment among infants up to 12 months, predominantly in aspects of communication and interpersonal relationships.^31^

In relation to the studies that used similar methodology but focused on populations with neurodevelopmental disorders, it is worth highlighting those described below.

A study of 506 Slovak families, 236 of whom had children diagnosed with ASD, monitored changes in children's behavior during the three waves of the COVID-19 pandemic. The findings showed an increase in inappropriate and maladaptive behaviors in both neurotypical children and children diagnosed with ASD, with minimal changes during the third wave in children with ASD, which could be explained by the greater availability of therapy during this period for such children.^6^

A longitudinal study, carried out in Australia, examined the impact of COVID-19 restrictions among 213 children aged 5 to 17 with attention deficit/hyperactivity disorder. Based on an online questionnaire completed by parents, it was found that compared to pre-pandemic, children had less exercise and less time outdoors, as well as less enjoyment of activities, while television, social media, and gaming time increased, with an increase in sad/depressed mood and loneliness. This study also highlighted parents' reports of positive changes for their children, including more family time.^32^

In the present study, the findings showed that the most significant risk factors associated with the perception of worsening neurodevelopment were the mother’s level of education, the child’s gender, and the child’s age group. Regarding the perception of abnormal neurodevelopment, the most significant associated factors were the child’s gender, age group, family participation in a social program, the family having been socially isolated during the COVID-19 pandemic, and losing a family member for COVID-19.

A previous study on risk factors for infant motor development showed that mothers with higher levels of education have more knowledge and concern about the adequate stimulation of their children, devoting more attention to their development.^33^ In this study, it was observed that the higher the level of maternal education correlated with greaterthe perception of worsening in the child’s neurodevelopment during the COVID-19 pandemic, corroborating the previous report.

Another study, with data collected at the beginning of the pandemic, found that parents and caregivers reported significant concerns about their children’s development during the pandemic. The study points to possible delays in cognitive development, difficulties in social interaction, and behavioral regressions. The environment of isolation and reduced external stimuli was identified as a determining factor. Besides, adverse life experiences related to family, environment, and society were considered environmental risks for problems in child development. Some of these adverse experiences include poor health conditions, lack of social and educational resources, maternal education, and family stress.^34^ In the present study, it was found that family participation in a social program, a factor that refers to restricted access to social resources, was a risk factor associated with the perception of neurodevelopmental abnormality. In other words, in this study, families participating in a social program reported a greater perception of abnormality than families not participating in a social program. Further, the present study showed a higher prevalence of perceived neurodevelopmental abnormality among respondents who said that they remained in social isolation, that is, they stayed at home with their children, which allowed them more time to observe and perceive them.

It is worth highlighting some limitations of this study, such as the heterogeneous socio-demographic distribution of the sample population, with a predominance of respondents from a more privileged social level among residents of Ceará. The sample refers to only two Brazilian states and, especially in the state of Ceará, includes respondents with a very high level of education in relation to the Brazilian population. This aspect may influence the perception of people with higher levels of education in a different way. Another limitation is that there was no precise information on previous diagnoses of neurodevelopmental disorders in the children under study. On the other hand, one of the strengths of this study is that the authors compared the respondents' perception with standardized scales and showed agreement at different levels.

In conclusion, this study found that parents' and/or caregivers' perception of normal neurodevelopment was significantly higher than their recognition of abnormalities. It also showed that these caregivers perceived a worsening in their children's neurodevelopment during the COVID-19 pandemic. Further, the risk factors for the perception of abnormal neurodevelopment were related to sex (male) age group (4-7 years), family socioeconomic level, degree of family isolation and the decrease of family members during the COVID-19 pandemic. For the perception of worsening in children's neurodevelopment, the risk factors found were the child being male, being in the 4-7 age group, and maternal schooling.

## Funding source

MLN is a researcher 1D supported by Conselho Nacional de Desenvolvimento Científico e Tecnológico (CNPq)- Brazil, PQ 303168/2021-8.

SRI received a scholarship from the Institutional Program to Encourage Postgraduate Studies - PRO-Stricto of the Pontifícia Universidade Católica do Rio Grande do Sul (PUC-RS).

## Conflicts of interest

The authors declare no conflicts of interest.
